# Microbes in Cahoots with Plants: MIST to Hit the Jackpot of Agricultural Productivity during Drought

**DOI:** 10.3390/ijms20071769

**Published:** 2019-04-10

**Authors:** Manoj Kaushal

**Affiliations:** International Institute of Tropical Agriculture (IITA), Mikocheni B, Dar es Salaam 34441, Tanzania; kaushal.mbg@gmail.com

**Keywords:** stomatal conductance, glomalin, strigolactones, miRNA, aquaporins, signalling

## Abstract

Drought conditions marked by water deficit impede plant growth thus causing recurrent decline in agricultural productivity. Presently, research efforts are focussed towards harnessing the potential of microbes to enhance crop production during drought. Microbial communities, such as arbuscular mycorrhizal fungi (AMF) and plant growth-promoting rhizobacteria (PGPR) buddy up with plants to boost crop productivity during drought via microbial induced systemic tolerance (MIST). The present review summarizes MIST mechanisms during drought comprised of modulation in phytohormonal profiles, sturdy antioxidant defence, osmotic grapnel, bacterial exopolysaccharides (EPS) or AMF glomalin production, volatile organic compounds (VOCs), expression of fungal aquaporins and stress responsive genes, which alters various physiological processes such as hydraulic conductance, transpiration rate, stomatal conductivity and photosynthesis in host plants. Molecular studies have revealed microbial induced differential expression of various genes such as *ERD15* (*Early Response to Dehydration 15*), *RAB18* (ABA-responsive gene) in *Arabidopsis*, *COX1* (regulates energy and carbohydrate metabolism), *PKDP* (protein kinase), *AP2-EREBP* (stress responsive pathway), *Hsp20*, *bZIP1* and *COC1* (chaperones in ABA signalling) in *Pseudomonas fluorescens* treated rice, *LbKT1*, *LbSKOR* (encoding potassium channels) in Lycium, *PtYUC3* and *PtYUC8* (IAA biosynthesis) in AMF inoculated *Poncirus*, *ADC*, *AIH*, *CPA*, *SPDS*, *SPMS* and *SAMDC* (polyamine biosynthesis) in PGPR inoculated *Arabidopsis*, 14-3-3 genes (*TFT1*-*TFT12* genes in ABA signalling pathways) in AMF treated *Solanum*, *ACO*, *ACS* (ethylene biosynthesis), jasmonate *MYC2* gene in chick pea, *PR1* (SA regulated gene), *pdf1.2* (JA marker genes) and *VSP1* (ethylene-response gene) in *Pseudomonas* treated *Arabidopsis* plants. Moreover, the key role of miRNAs in MIST has also been recorded in *Pseudomonas putida* RA treated chick pea plants.

## 1. Introduction

**Figure 1:**
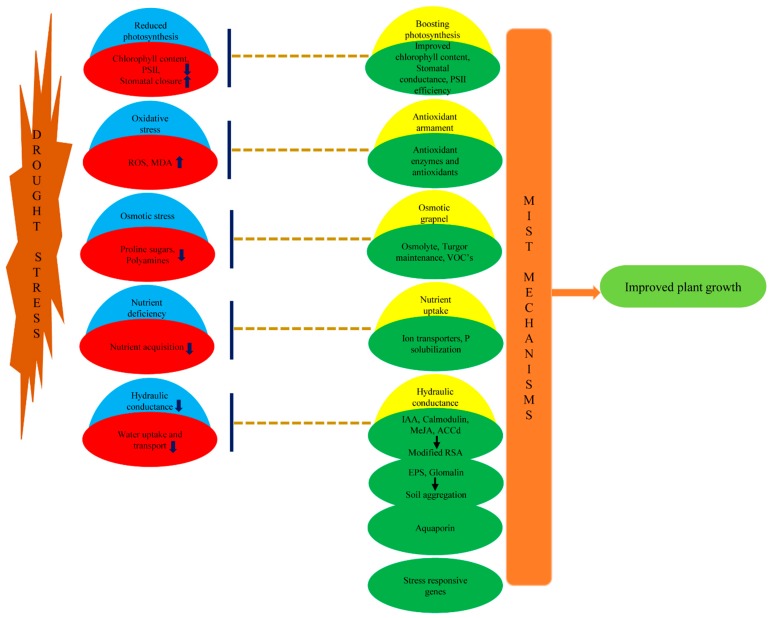
On the left side drought stress negatively impacting plant growth by secondary osmotic, oxidative stresses, reduced photosynthesis, hydraulic and nutrient conductivity. On the right side major microbial induced systemic tolerance (MIST) mechanisms displayed by microbes enhancing plant growth during drought stress.

Escalation in drought incidences as a result of climate change meddles in agricultural productivity as water deficit conditions during drought interfere with the cellular metabolic machinery of plants [1]. Water deficit during drought reduces soil water potential resulting in cell dehydration and increased reactive oxygen species (ROS) ultimately inducing secondary stresses such as osmotic and oxidative stress hampering plant growth. Water deficit results in stomatal closure, reduction of turgor pressure, chlorophyll (Chl) content and photosynthesis [2]. Integrating microbes as component of agricultural system for enhanced drought tolerance in plants needs to be endorsed to boost sustainable crop production. Microbes can enhance drought tolerance in plants via microbial induced systemic tolerance (MIST) mechanisms involving various biochemical, physiological and molecular modifications in host plants. MIST mechanisms basically comprise changes in hormonal profiles affecting root system [3], stomatal conductivity [4], photosynthesis [5], enhancing nutritional status [6,7], osmotic grapnel [8], sturdy antioxidant armament [9,10], bacterial exopolysaccharides (EPS) [11] or fungal glomalin production [12] and differential expression of stress responsive genes [11] (Figure 1). In similar direction key role of aquaporins in AMF inoculated plants and volatile organic compounds (VOCs) in PGPR treated plants has also been established (Figure 1). Primary physiological criteria examined during water deficit includes relative water content (RWC), stomatal conductance (*gs*), Chl content, photosynthetic rate (*Pn*), *Fv*/*Fm* (ratio of variable to maximal fluorescence) and malondialdehyde (MDA) content in leaves [13]. It has been observed that plant growth-promoting rhizobacteria (PGPR) inoculation maintains the RWC [14], *gs* [5], Chl content [9], photosystem II efficiency [15], *Pn* [16] in plants during drought stress conditions. Similarly, arbuscular mycorrhizal fungi (AMF) inoculation have been observed to increase drought tolerance via maintaining *Pn* [17], RWC [18,19], MDA content [20], *Fv*/*Fm* [21] and *gs* in host plants [22]. This is primarily accomplished via enhanced hydration status and nutritional profile preserving the cellular turgor and machinery in plants during drought. There are reviews reporting PGPR induced drought tolerance referred as rhizobacterial induced drought endurance and resilience (RIDER) [23] or induced systemic tolerance (IST) [24,25,26] and AMF induced drought tolerance [27].

In present review article, we address the various MIST mechanisms responsible for enhancing plant productivity during drought as a result of interplay between plant and microbes (both PGPR and AMF).

## 2. Microbe Mediated Biochemical and Metabolic Mechanisms to Regulate Oxidative and Osmotic Stress

### 2.1. Osmotic Grapnel for Turgor Restoration

**Figure 2:**
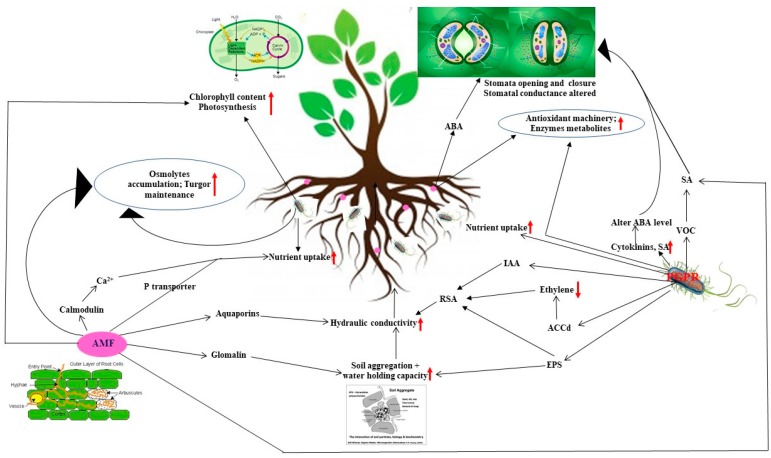
Schematic representation displaying modulation of various plant biochemical and physiological processes in host plants as microbes (AMF and PGPR) buddy up with plants to alleviate drought stress.

Abiotic stresses can alter the osmolality levels in plant which impedes its growth and survival. Hence to counteract it, osmotic adjustment and conglomeration of compatible solute accumulation is a requisite to combat dehydration loss caused by osmotic stress during drought. Plants under water stress can survive either by reducing water loss or sustaining water uptake. Osmotic adjustment is defined as a biochemical mechanism that involves the amassment of compatible osmolytes or solutes like proline, sugars, polyamines, betaines, quaternary ammonium compounds, polyhydric alcohols, amino acids and water stress proteins like dehydrins. Compatible osmolytes aggregation lowers cell water potential thus averting water loss and maintaining turgor in metabolically rustling cells which shields enzymes, proteins and biomembranes against oxidative stress [18]. Osmolyte production by microbes (PGPR or AMF) and plant osmolytes during water deficit act concertedly to mitigate cell turgidity damages [10] (Figure 2). Proline a proteinogenic amino acid is imperative for primary metabolic processes during osmotic stress as it acts a molecular chaperone by quenching ROS that reduces lipid peroxidation, modifying cytosolic acidity and preserving proteins and membranes. Priming plants either with PGPR or AMF modifies proline levels in plants ensuring plant survival during drought. Various research studies have reported increase in levels of proline as a result of rhizobacterial inoculation in plants [14,28,29]. Not only rhizobacterial inoculation but AMF inoculation also contributes towards osmotic adjustment [30]. It was observed that the higher levels of flavonoids and proline in AMF (*Claroideoglomus etunicatum*) colonized *Pistacia vera* seedlings in comparison to non-mycorrhizal inoculated plants that helped plants to survive during drought [20]. A 3 fold and 2 fold increase in proline levels was recorded in leaves and roots respectively in AMF inoculated bell pepper that sustained tissue water status [31]. Proline accumulation has also been observed in other AMF inoculated plants viz. *Lactuca* [32], *Macademia* [33], white clover [34], maize [35], *Loquat* [8], *Allium* [36] and rice [37]. Raised proline levels in AMF inoculated plants ensures plants more resilience and adaptability to cope up osmotic stress. In contrast some research studies have reported decreased levels of proline in AMF inoculated plants viz. *Erythrina* [38], *Knautia* [39] and *Poncirus trifoliata* [40] and *Cyclobalanopsis* [41]. In addition AMF inoculated *Glycine* plants showed elevated proline aggregation in roots and reduced proline content in shoots during water deficit [30]. It was observed that lower proline levels showed meagre injury in AMF inoculated plants during drought stress [42]. Trehalose is another osmoprotectant that has key role in cell signalling, stabilization of cell structures and proteins [25]. Macro array analysis of 7200 EST from *P. vulgaris* nodules primed with *Rhizobium etli* overexpressing trehalose-6-phosphate synthase gene showed upregulation of genes engaged in stress tolerance in comparison to non-inoculated counterparts [43]. Similar observations were recorded in *A. brasilense* primed maize plants during drought [44]. Glycinebetaine as an osmoprotectant has pivotal role in stabilizing proteins enzymes and membranes during abiotic stresses. Elevated levels of glycine betaine and choline conferred drought tolerance to *Bacillus subtilis* inoculated *Arabidopsis* plants as compared to its xipotl mutant [45]. Various researchers has confirmed the osmoprotective role of sugars by AM inoculation during drought stress [30,46]. It was observed that the raised soluble sugar content in mycorrhizal inoculated sweet potato, having more aggregation in roots rather than shoots [19]. Polyamines such as cadaverine, spermidine (Spd), spermine (Spm) and putrescine (Put) are major metabolites that can enhance osmotic tolerance to drought in host plants in addition to a key role in cell differentiation, root elongation and transcriptional regulation. Research studies confirmed role of cadaverine in increasing root growth of rice seedlings primed with *Azospirillum brasilense* during osmotic stress [47]. Increase in cellular levels of polyamines was observed in *Arabidopsis* plants treated with the Spd-producing *Bacillus megaterium BOFC15*. Inoculated plants survived drought stress because of robust root system possessing longer primary roots and increased lateral roots in comparison to the control plants [48]. Some studies have suggested correlation among polyamines, abscisic acid (ABA) and abiotic stresses [48,49] hence some research can be carried forward in this direction to unravel the MIST mechanisms involved drought stress.

### 2.2. Antioxidant Armament to Downstream Oxidative Stress

Usually ROS production as metabolic products is minimal in plants growing under normal conditions. Skewness induced as a result of ROS overproduction and detoxification in plants as a consequence of increased photorespiration and disrupted photosynthetic machinery is a cardinal change during water deficit or drought stress. ROS such as superoxide anion radical, hydrogen peroxide, hydroxyl radicals and singlet oxygen causes oxidative stress in plants leading to catastrophic damages to lipids, proteins and nucleic acids. Despite the fact that raised ROS levels induce oxidative damages, their lower levels are essential for the plant as they are involved in signalling events that activates various defensive pathways. Hence it is essential to manage ROS via coordinated control of ROS generation and ROS quenching systems to control oxidative stress damages and signalling functions. Plants are rigged with intrinsic setup of antioxidant defensive network to quash excess ROS. Antioxidant gadgetry constitutes enzymatic group (superoxide dismutase (SOD), peroxidase (POX), ascorbate peroxidase (APX), glutathione reductase (GR), catalase (CAT), ascorbate peroxidase (APX) and guaiacol peroxidase (GPOD)) and non-enzymatic components (glutathione, ascorbate, flavonoids, carotenoids and tocopherols) [18]. Even though plants are armed with robust antioxidant defensive machinery, the decrease in plant productivity have been reported that marks the drought stress significance in alteration or malfunctioning of enzymatic activity. Pioneer research studies displayed enhanced plant protection via microbial colonization or inoculation. AM symbiosis or rhizobial inoculation simulates highly robust ROS-quenching system in plants hence preserving membrane integrity and protein stability during oxidative stress [50] (Figure 1). Various research activities focussed on drought stress involves measurement of antioxidant enzymes so as to assess the oxidative damage. It is observed that treatment of plants with AM amends antioxidant enzyme levels, reducing ROS and lipid peroxidation of membranes [51]. Polyunsaturated fatty acids of membrane phospholipids are the prime targets of ROS during water deficit initiating lipid peroxidation and fatty acid degradation that generates various cytotoxic products such as MDA. Quantification or measurement of MDA content is a used a biomarker of oxidative stress induced lipid peroxidation. BBS (*Bacillus cereus* AR156, *Bacillus subtilis* SM21 and *Serratia* spp. XY21) primed cucumber plants displayed lower MDA levels and decreased relative electrical conductivity in leaves in comparison to control plants [9]. Identical observations of reduced MDA content were recorded in *A. brasilense* inoculated *Arabidopsis* plants during drought [5]. Decreased content of MDA was also measured in chickpea colonized with *Glomus* species during drought stress [52]. Similar decrease in MDA levels was recorded in AM fungi treated date palm [53], maize [35] and pistachio plants [20]. Various research studies have reported alterations in levels of antioxidant enzymes in plants by PGPR inoculation as a major MIST mechanism against drought [5,6,10,11,54,55,56,57]. It was observed that the elevation in SOD and *cAPX* transcription levels in BBS consortium primed cucumber plants that abated oxidative stress damages [12]. Increased activity of POX and SOD over a period of 15–43 days was noticed in *Pseudomonas* treated mung bean plants however CAT activity first inflated over 15–36 days and later decreased [57]. In contrast *Pseudomonas putida GAP-P45* inoculation downsized APX, CAT, GPX contents in maize plants in contrast to uninoculated counterparts [58]. Identical observations with decreased GR and APX activity were recorded in *Lavandula* plants treated with Bacillus thuringiensis [10]. Higher activity of CAT, APX and reduced MDA level was observed in strawberry inoculated with *F. mosseae* [59]. It was observed that AM symbiosis enhanced drought tolerance in rice plants via aggregation of glutathione that decreased hydrogen peroxide ultimately improving photosynthetic activity [60]. Mycorrhiza inoculated pistachio seedlings displayed increased levels of soluble sugars, flavonoids and POX activity in comparison to control plants under water stress conditions [20]. Mycorrhizal inoculation of lettuce improved plant stress tolerance due to boosted aggregation of antioxidants viz. carotenoids and anthocyanins in leaves during water deficit conditions [61]. Dual inoculation of flax plants with AM and *Pseudomonas* showed higher aggregation of enzymatic and non-enzymatic antioxidants compared to plants inoculated singly either with PGPR or AM [62]. *Rhizophagus irregularis* inoculated *Robinia* seedlings had raised activity of SOD, POD, CAT, APX and GR activities and higher transcript levels of Cu/Zn-SOD during drought stress [63]. Ascorbate and glutathione levels were recorded higher in *Poncirus trifoliata* trees colonized by *Glomus versiforme* in contrast to uninoculated trees during a drought period of 12 days [64]. Research studies in mycorrhizal (*Rhizophagus intraradices*) inoculated *Eleusine* seedlings revealed 16% higher flavonoid content and 25% higher ascorbate level in the leaves during drought stress [65]. In addition glutathione content was also recorded to be higher in AM inoculated seedlings.

## 3. Mechanisms Affecting Plant Physiology to Cope Drought

### 3.1. Improved Soil Structure Ameliorating Plant Water Stature Affecting Stomatal Conductance and Boosting Photosynthesis

The altered physical and biological properties of soil due to water deficit impedes the plant growth. Among the chief physiological mechanisms for upgraded MIST in plants during water deficit conditions could be attributed to boost in hydraulic conductivity of roots or water relations. Extraradical hyphae of AMF are engaged in water uptake and nutrient translocation [66] which is more significant during water deficit conditions as these hyphae are capable of invading soil pores that are impervious to root hairs. The movement of water through AMF hyphae is apoplastic as water absorbed by the extraradical hyphae from the soil is transferred to cortical apoplast which then adjoins water passage through root apoplastic pathway [67]. Extensive root system of AMF hyphae increase the exposure area in soil around plant host which directly alters relative water potential, RWC [68] and transpiration rate in plants [69]. Moreover glomalin formation as a result of AMF colonisation also affects water relations during drought stress [70,71]. Glomalin quantified as glomalin-related soil protein (GRSP) is a glycoprotein formed by AMF spores and hyphae in soil and roots. It acts a glue which is involved in soil aggregate formation, carbon sequestration and water retention [72]. AMF have the ability to structure microchannels or regulates the connectivity of pore spaces, that serves as a close water passage across air zones with direct connection to roots [73]. An elevation in level of GRSP and aggregate stability was observed in AMF colonized orange seedlings during drought conditions [71]. Similar observations of elevated total GRSP (T-GRSP), easily extractable GRSP (EE-GRSP) and leaf/soil water potential were recorded in AMF inoculated *Citrus tangerina* seedlings than non-AMF seedlings during water deficit [74]. As glomalin production by AMF improves soil structure, in similar fashion EPS production by rhizobacterial strains also plays pivotal role in enhancing plant growth under water deficit via formation of hydrophilic biofilms around plant roots protecting them from soil drying hardness [16,75] (Figure 2). Numerous research studies have documented EPS mediated MIST in PGPR inoculated plants such as sunflower [76], maize [14,56], wheat [11,77] however water holding capacity (WHC) varies according to polysaccharide components present in EPS. MIST via EPS can alleviate drought stress as it boosts root-adhering soil/root tissue ratio, RAS macroporosity and macroaggregate stability [14] augmenting water and nutrient uptake from rhizospheric soil thus sustaining higher water potential in vicinity of roots. AM colonization affects the aboveground physiological processes by lowering water potential in leaves and xylem in response to reduced soil water potential which modifies stomatal conductance, transpiration rate, hydraulic conductivity and photosynthetic efficiency during water deficit conditions [67]. Water deficit decreases soil water levels causing stomatal closure which in turn decreases photosynthesis, however AMF colonisation increases photosynthesis by improving water stature as increased stomatal conductivity results in higher CO_2_ diffusion in the mesophyll cells [78]. Increased stomatal conductance in AM colonized plants have been confirmed in rosemary [79] and rice [37] under water deficit. In contrary, AM colonized white clover (*Trifolium repens* L.) showed reduced stomatal conductance and higher RWC [80] contributing towards enhanced water use efficiency (WUE). Shifts in ABA levels increases the drought tolerance in AM plants [81] as ABA alters the plant water stature via regulating transpiration rate, stomatal conductance and induction of stress tolerance genes. ABA content was found to be higher in non AMF plants as compared to AMF plants indicating less drought stress experienced by non AMF plants [39], however variations in stomatal conductivity and photosynthesis depend on host and fungal species. ABA pivotal role among non-nutritional mechanisms affecting stomatal conductance during drought in AM plants has been suggested [82] and will be discussed later in phytohormone section. It was confirmed that ABA independent stomatal closure in *P. chlororaphis* O6 treated plants [83]. Reduced stomatal apertures were observed in inoculated wild-type as well as ABA-insensitive *Arabidopsis* mutant plants that reduced transpiration levels thus enhancing MIST to drought in host plants. ROS production damages the photosynthetic machinery hence reducing photosynthesis during water deficit, however microbial inoculation can enhance tolerance in host plants as it improves plant water stature which boosts photosynthesis via increased stomatal conductance [78]. Increased photosynthesis in rhizobacterial or AMF inoculated plants can be attributed to increase in photosynthetic pigments and more efficient photosynthetic machinery [84]. Elevation in photosynthetic pigments in AMF treated plants during water deficit has been recorded in the *Tagetes erecta* L. [84], *Erythrina variegata* Linn. [38], *Ipomoea batatas* (L.) Lam. [19] and citrus [85]. Moreover, decreased content of sugars and starch was observed in addition to elevated level of photosynthetic pigments (chlorophyll and carotenoids) in AMF *Glomus versiforme* inoculated *Poncirus* [17] and *F. Mosseae* colonized *Erythrina* hence confirming sugar removal to enhance sink strength of AMF fungi via increased photosynthesis.

### 3.2. Modification of Hormonal Contents

Phytohormonal alterations in microbial colonized host plants ensures plant survival during drought stress. Water deficit caused by drought negatively affects the root metabolism and photosynthetic activity in plants. It is observed that PGPR treated plants display remodelled root system architecture (RSA) comprised of increased root tips and surface area [86,87] ultimately boosting hydraulic and nutrient conductance during stress [24] (Figure 2). Auxins or indole acetic acid (IAA) plays crucial role in regulating plant growth as they are responsible for cell division in shoots, apical dominance and root branching. Alteration in IAA levels in rhizobacterial treated plants led to reduced leaf water potential, raised leaf water levels in *Azospirillum* inoculated wheat plants [88] and increased uptake of water in PGPR or AMF treated clover plants that enhanced osmotic stress tolerance of host plants [89,90]. Wheat plants primed with *Bacillus thuringiensis* showed increase in length and density of root hairs and lateral roots by modifying hormonal contents due to production of IAA and 1-aminocyclopropane-1-carboxylate deaminase (ACCd) by rhizobacteria [11]. IAA production by rhizobacteria increased drought tolerance of *B. thuringiensis* inoculated *Lavandula* plants by restoring the various physiological and metabolic processes of the host plant [10]. Higher levels of IAA and ABA were recorded in AM symbiotic *Phaseolus* plants in contrast to non AM plants under both well-watered and drought stress conditions [91]. Similar results of raised levels of IAA, methyl jasmonate and calmodulin were observed in roots of mycorrhizal trifoliate orange which supported the fact of superior RSA in AM plants during drought or water deficit. Modified RSA by AM inoculation increased root hair length and density under drought stress, however no significant changes were observed in diameter of root hairs [3]. Gibberellins play important role in plant growth as they are involved in stem elongation, germination, flowering, senescence and fruit ripening. Studies conducted by different researchers have indicated that inoculation by GA producing bacteria also alleviates drought stress effects in host plants. A significant rise in GAs was observed in *Azospirillum lipoferum* primed maize plants [92], rhizobacterial consortia (*Burkholdera cepacia SE4*, *Promicromonospora* spp. *SE188* and *Acinetobacter calcoaceticus SE370*) colonized cucumber plants [55] and *Pseudomonas putida* treated soybean plants [93]. Cytokinins has pivotal role in process of cell division, shoot growth and maintains photosynthetic activity, stomatal opening during drought. Moreover positive effect of cytokinins on plant survival have been also observed in PGPR treated plants during drought stress conditions [94,95] (Figure 2). Rapid biosynthesis of ABA as a major physiological response or non-nutritional mechanism in response to drought is well documented in various studies [81]. Its biosynthesis in plant roots and then translocation to leaves starts the drought tolerance process via stomatal regulation. Various studies have recorded the modifications in the ABA content via microbial colonization results in enhanced tolerance during water deficit conditions [81,96]. Plant water stature in plants gets altered as a result of ABA level shifts by regulating root hydraulic conductance, transpiration rate and inducing expression of genes involved in drought tolerance. Research studies have revealed AM symbiosis or rhizobacterial inoculation induced shifts in ABA level affects various physiological processes including stomatal conductance [4,82,95] (Figure 2). Contrary observations were recorded in ABA levels in AMF treated plants which can be correlated to AMF genotype thus pointing towards complex plant-microbe interactions [82]. Decreased ABA content in AMF plants led to increased transpiration and water uptake by host roots [96] however elevation in ABA level was recorded in AMF inoculated or non-inoculated tomato plants during drought stress [97] indicating water deficit induced ABA regulation in AMF plants. A systemic reduction in ABA levels was observed in the whole root system of AMF (*Rhizophagus intraradices*) treated maize plants subjected to drought stress. Elevation in ABA levels in response to drought response was observed in non AM plants in contrast to AM plants indicating less stress experienced by AM plants [39]. Significant rise in ABA levels was reported in *A. brasilense* inoculated maize plants [92], *Phyllobacterium* inoculated *Arabidopsis* [4], *Bacillus* treated *Platycladus orientalis* seedlings [95] thus modifying root system architecture by increasing lateral roots [5], reducing leaf transpiration [4] and increasing stomatal conductance [95] hence conferring MIST to host plants. However contrasting results with decreased ABA levels were recorded in PGPR inoculated cucumber [55] and soybean plants [93] during drought and salinity stress pointing towards less stress experienced by inoculated pants in compared to non-inoculated ones. Jasmonic acid (JA) and its methyl ester, methyl jasmonates (MeJAs) play pivotal role affecting plant growth and physiology during normal or stress conditions [98]. JA signalling pathway gets activated in response to colonization by AMF and JA participates in stress signalling pathways via ABA levels in host plants [97]. An elevation of carbohydrate content from shoots to roots causes variations in osmotic potential of roots which increases JA levels. Alleviation of oxidative stress via production of JA and salicylic acid (SA) was observed [99]. A significant surge in JA and SA levels was also recorded in *P. putida* primed soybean plants [93]. JA and MeJAs play pivotal role affecting plant growth and physiology during normal or stress conditions [98]. Increase in levels of MeJA was observed in AM inoculated barley [100], soybean [101] and trifoliate orange plants [3,102] during water stress conditions having positive correlation to root hair density and vice versa related to root hair length thus indicating MIST in AM inoculated plants. Biosynthesis of ethylene is enhanced during abiotic stresses such as drought and salinity and its levels above threshold value retards plant growth via affecting root development, inhibiting seed germination and causing senescence. It is observed that ethylene precursor, 1-aminocyclopropane-1-carboxylate (ACC) is released in rhizospheric vicinity of host, then ACCd possessing rhizobacterial strains cleaves ACC to ammonia and a-ketobutyrate, thus decreasing ethylene levels which in turn promotes plant growth [103]. Plant growth promotion by various ACCd PGPR strains have been confirmed by various researchers during drought [104,105,106,107] (Figure 2). Reduced ethylene levels were recorded in *Bacillus licheniformis K11* treated pepper [107] *Pseudomonas* primed *Pisum* plants [104] which induced better root development that increased plant water and mineral uptake ultimately inducing MIST in host plants to drought stress. Lower emission of ethylene was observed in leaves and roots of velvet bean plants inoculated with ACCd rhizobacteria which improved root and shoot length and biomass ultimately boosting plant performance during drought stress [108]. A significant increase in soil moisture and the root adhering soil/root tissue ratio was observed in foxtail millet inoculated by *Pseudomonas fluorescens* DR7 which possessed ACCd and EPS production ability [109]. There have been no reports regarding the role of phytohormone brassinosteroids in relation to MIST to drought in plants which needs to be explored as BRs role in mitigation of stress have been confirmed in various research studies [110]. Strigolactones are newly identified phytohormones derived from carotenoids affecting RSA, reproductive development and signalling processes [111]. A significant correlation has been reported between ABA production and strigolactones in AMF treated plants during stress conditions [96] as ABA regulates strigolactones synthesis [112]. It was reported that strigolactones biosynthesis induced drought tolerance in lettuce and tomato [113]. Future research studies can be focussed on how ABA and strigolactones profiles are altered in microbial treated plants during drought stress and their possible role in alleviating negative plant growth effects caused by water deficit.

### 3.3. Accelerated Nutrient Acquisition

Drought affects diffusion and mobility of nutrients in soil due to poor soil structure as a result of water deficit. *Cucumis* plants inoculated with PGPR strains subjected to drought showed increased levels of P and K ions in comparison to control plants [55]. Nitrogen content in leaves of pea plants treated with *V. Paradoxus* 5C-2 was observed as inoculation increased nodulation process [106]. A significant rise in level of macronutrients (K^+^, Ca^2+^ and Mg^2+^) and micronutrients (Zn^2+^, Mn^2+^ and Cu^2+^) was recorded in shoots of *Lavandula* plants primed with *B. thuringiensis*. Raise in K^+^ levels reduced stomatal conductance necessary for sustaining turgor pressure during drought conditions [10]. Major reason behind enhanced nutrient status in AM plant is increased absorption surface of extraradical hyphae for expansive exploration of nutrients from soil. The extraradical hyphae of AMF gets extended deep inside rhizospheric soil for absorption of nutrients from there they are transported to arbuscules in cortical cells and are finally released into the apoplast to ameliorate nutrient deficiency caused by stress [6,20,114,115]. Water deficit decreases P acquisition, however, improve AM symbiosis P nutrition affecting the water status in plants hence increasing host growth rates during drought. A significant decrease in P content was observed in non AM plants of citrus [85] and marigold [84] in comparison to AM plants. Increased P nutrition in AM plants can be attributed to higher phosphatase activity and absorption of P, N, K, Ca, Zn and Cu by extraradical hyphae [20,116]. *G. Versiforme* treated trifoliate orange seedlings displayed increased K, Ca in leaves and P, Ca and iron levels in host roots during water deficit conditions [117]. Similar increase in levels of nutrients such as P, K, Zn and Mn was recorded in *Pistachio* plants colonized by *G. intraradices* and *G. mosseae* [114]. AMF plants displayed a positive correlation between aquaporins and enhanced N content as it was involved in transportation of ammonium [118]. Research studies carried out in AM colonized ryegrass revealed enhanced plant fitness during drought stress due to increased N uptake and boosted activities of N-assimilating enzymes that led to rise in amino acids and protein levels [119]. It was reported that enhanced nutritional value as a result of AM (*Rhizophagus intraradices*) inoculation conferred drought tolerance to host *Zea* plants [120]. AM inoculation led to significant increase in contents of P, N and Mg which boosted stomatal conductance, pigment (chlorophyll) content and photosynthetic efficiency of host plants under water stress [60,85]. Colonization of *Trifolium* plants by PGPR strains and AM raised the K levels (217% in *Bacillus thuringiensis* + AM and 348% in *Pseudomonas putida* + AM or *Bacillus megaterium* + *Rhizophagus intraradices*) which helped plant to cope drought stress as K affects the physiological processes such as water uptake and photosynthesis. In addition a significant increase of 89%, 131%, 79% and 62% in P, Ca, Mg, Zn was observed respectively in plants inoculated with the autochthonous bacteria and mycorrhiza [6]. Studies on lettuce plants primed by *Bacillus* sp. and *Klebsiella* showed increased P, K contents and more biomass production in bacterial treated plants in comparison to AM inoculated ones which was associated with uptake of macronutrient (Ca, K, Mg and P) [7]. Increase in root influx of Ca^2+^ was noticed in AMF trifoliate orange seedlings during well-watered and water deficit conditions which was due to boosted Calmodulin (CaM), a calcium sensor synthesis as it was able to bind more calcium ions. CaM levels induced as a result of AMF inoculation were related to SOD and CAT activities enhancing MIST in host plants to drought [74] (Figure 2). In future research should be focussed on unravelling the signalling pathways behind CaM mediated antioxidant defence to drought in microbial inoculated plants.

### 3.4. Volatile Organic Compounds (VOCs) in PGPR Inoculated Plants

VOCs (2R, 3R-butanediol and 3-hydroxy-2-butanone) production by PGPR strains can alter the expression of genes related to cell wall structure [121] and auxin homeostasis [122] in *Arabidopsis* plants. Synthesis of choline and glycine betaine was induced due to VOCs production in *Arabidopsis* plants conferring osmotic stress tolerance [123]. *Pseudomonas chlororaphis* O6 generated 2R, 3R-butanediol (VOC) induced stomatal closure in *Arabidopsis* plants thus contributing towards MIST in plants [124] (Figure 2). It was reported that the plant VOC production was altered during PGPR inoculation which enhanced drought tolerance in wheat plants [11]. Benzaldehyde, b-pinene and geranyl acetone levels were boosted with increased drought stress but *B. thuringiensis* colonized wheat stressed plants showed reduced emission of these VOCs in comparison to non-primed stressed counterparts.

## 4. Molecular Mechanisms to Encounter Water Deficit

### 4.1. AM Induced Expression of Water Transporter Aquaporins

Expression of abiotic stress responsive genes as a result of AMF inoculation in plants alters various physiological, biochemical and metabolic pathways in host plants conferring drought tolerance. Water deficit conditions can be tackled by increased water absorption or transportation from the soil or through root tip to plants via extra radical mycelium of AMF. Hence water equilibrium during drought in plants is sustained either by process of diffusion or through aquaporins, however water transport via latter is 10–100 times higher [125]. Various research studies involving molecular techniques have revealed important role of regulatory genes responsible for encoding water transport components such as aquaporins genes in AMF plants during drought conditions [126,127]. Aquaporins are transmembrane proteins belonging to the Major Intrinsic Protein (MIP) superfamily encoded by aquaporin genes involved in rapid water transport and diffusion of various physiological substrates viz. CO_2_, silicon and ammonia. Expression of aquaporin genes may be upregulated or downregulated by AMF symbiosis to reduce transpiration rate or to enhance leaf water potential and hydraulic conductivity in roots of host plants [125,126,128,129] (Figure 2). The gene expression of aquaporins in AMF and non-AMF plants was analysed [130]. It was observed that three genes displayed differential regulation. A meagre inhibition in AMF and no change in expression pattern in non AMF plants was observed for PIP1;1, however similar inhibition in expression pattern of PIP1;2 was recorded during drought. Moreover induction of PIP1;3 was observed in non AMF plants but suppressed in AMF plants, thus concluding that expression of aquaporins during drought stress varies according to AM species and specific aquaporins in AMF plants. Inhibited expression patterns of aquaporins during water deficit can be an approach of water conservation in AMF plants to maintain required water content [125]. Expression of aquaporin genes GintAQPF1 and GintAQPF1 cloned from *R. irregularis* was upregulated in cells of cortex and extraradical mycelia of maize roots inoculated by AMF (*G. intraradices*), clearly indicating role of AMF aquaporins in enhancing water transport through AMF hyphae to plant. Researchers utilized suppression subtractive hybridization (SSH) to identify various genes involved in drought tolerance in AMF colonized *Cajanus cajan* [131]. Among 182 expressed sequence tags (ESTs) procured by sequencing 102 were up regulated and 40 were down regulated. Moreover 35 differentially expressed genes obtained by RT PCR were related to impart drought tolerance in AMF inoculated plants. Studies highlighted the regulation of aquaporins by AMF (*R. irregularis*) inoculation in maize plants for drought stress alleviation [126]. It was observed that AMF colonization regulates expression of 16 among total of 36 aquaporins, however expression varied in accordance to severity and time period of drought stress. A significant increase in expression of aquaporin genes *RpTIP2;1* and *RpPIP2;1* in roots, stem and leaves was observed in *Rhizophagus irregularis* treated *Robinia* plants which enhanced water flow inside plant tissues mitigating drought stress effects [132]. Moreover contrasting correlation between various aquaporins and hydraulic conductance in roots was observed in mycorrhizal plants in comparison to non-mycorrhizal plants that abated oxidative and osmotic stress in inoculated plants. Increased expression of *OePIP1;2* and *OeTIP1;2* led to increased hydraulic conductivity however vice versa was observed for *OePIP1;3*, *OePIP2;4* and *OeTIP1;3* [133]. In another study different expression of aquaporins was observed in drought sensitive(ds) and drought tolerant(dt) cultivars of maize, where AM colonized ds plants enjoyed more physiological benefits. A downregulation of *ZmPIP1;1*, *ZmPIP1;3*, *ZmPIP1;4*, *ZmPIP1;6*, *ZmPIP2;2*, *ZmPIP2;4*, *ZmTIP1;1* and *ZmTIP2*; and upregulation of *ZmTIP4;1* genes was observed in AM colonized ds plants, hence concluding that downregulated expression of genes in ds cultivar is a possible way to reduce water deficit induced stress changes [134]. Transcriptomic analysis revealed the upregulation of two putative-aquaporin genes *LjNIP1* and *LjXIP1* during AM symbiosis in roots of *Lotus japonicus*, however both of them related to different families of plant aquaporins. It was observed through laser microdissection that *LjNIP1* having putative function as an aquaporin was expressed principally in arbuscule-containing cells. LjNIP1 and LjXIP1 both were unable to transport solutes like ammonium or urea however LjNIP1 was capable of transporting water [129]. It has been established that LjNIP1 has important role in maintaining cell turgor either through water paths within the inner membrane systems or via water passage entry from the fungus to the host plant [135]. Gene expression analysis done in tomato plants colonized by two AM fungi (*Funneliformis mosseae* and *Rhizophagus intraradices*) under the water stress condition revealed different transcriptional patterns of 3 aquaporin genes in roots corresponding to different subfamilies viz. NIPs (NOD26-like intrinsic proteins), TIPs (tonoplast intrinsic proteins) and PIPs (plasma membrane intrinsic proteins. Expression of *LeNIP3* was upregulated in AM colonized plants especially treated with *F. mosseae*; however both *LePIP1;1* and *LeTIP2;3* were observed to be down-regulated in AM colonized as well as control plants during water stress [136]. However contrary results were recorded in *Lactuca* and *Glycine max* plants colonized with *F. mosseae* and *R. irregularis*, significant decrease in expression of *PIP* genes was noticed in AMF plants in comparison to non AMF plants during water deficient conditions corresponding to increased RWC. Downregulation of aquaporin genes as a result of AM symbiosis was observed as a mechanism to control the water loss from the cells during drought [137]. Reduced expression of *GintAQP1* gene in lettuce plants was observed during drought and was not even changed with increased AM colonization of host roots [138]. These contrasting results in expression of aquaporins illustrates differential mechanisms for altering the hydraulic conductivity and abstaining water loss.

### 4.2. Insights in Rhizobacterial and AM Induced Expression of Genes

Molecular studies have revealed that microbial treated or colonized plants display different expression of genes in comparison to non-colonized plants ultimately enhancing drought tolerance (Figure 1). MIST to drought was observed in *Arabidopsis* plants primed with *Paenibacillus polymyxa* due to induction of drought stress responsive genes, *ERD15* (*Early Response to Dehydration 15*) and ABA-responsive gene, *RAB18* (*LEA*) [139]. BBS consortium displayed primary role in eliciting MIST to drought stress in inoculated cucumber plants via sustaining the transcriptional levels of *cAPX* (cytosolic ascorbate peroxidase), rbcS and rbcL (RuBisCO small and large subunits) in leaves ultimately magnifying antioxidant and photosynthetic machinery of hosts to overcome drought [9]. A significant upsurge in transcripts of defence related genes *PR1* (SA regulated gene), *pdf1.2* (JA marker genes) and *VSP1* (ethylene-response gene) was noticed during transcriptosome analysis carried in *Arabidopsis thaliana* plants colonized by *Pseudomonas chlororaphis O6* thus confirming MIST to drought stress [83]. Gene profile studies revealed over expression of *Cadhn* (dehydrin-like protein), chaperones *sHSP* (Plant small heat shock proteins), *VA* (vacuolar H^+^ ATPase) and *CaPR-10* (Pathogenesis-related protein 10) in *Bacillus licheniformis K11* treated pepper plants [107]. Upregulated expression of drought responsive genes *DREB2A* (Dehydration responsive element binding), *DHN* (Dehydrin) was recorded in *Vigna* plants treated with *Pseudomonas aeruginosa GGRJ21* in comparison to control plants [57]. The expression analysis of 11 stress responsive genes was investigated in *Pseudomonas putida MTCC5279* inoculated chick pea exposed to drought stress. Quantitative real-time qRT-PCR analysis revealed upregulation of transcription factor *DREB1* (Dehydration responsive element binding gene) and *NAC1* in uninoculated plants however PGPR inoculation downregulated its expression in both cultivars, both TFs are primarily involved in abiotic stress tolerance however NAC is also related to some developmental pathways. PGPR inoculation repressed expression of stress responsive *LEA* and dehydrins that are involved in protecting macromolecules. A significant decrease in expression of genes encoding ROS scavenging enzymes (CAT, APX, GST) and ethylene biosynthesis genes namely *ACO* and *ACS* by inoculated plants confirmed ability of PGPR strains to MIST via restoration of normal growth conditions. In addition upregulation of jasmonate *MYC2* gene was observed in desi cultivar of chickpea establishing role of JA in drought stress tolerance [140]. Inoculation of wheat plants with strains *Bacillus amyloliquefaciens 5113* and *Azospirillum brasilense* NO40 eventually caused upregulated expression of stress genes such as APX1 (ascorbate peroxidise), SAMS1 (S-adenosyl-methionine synthetase involved in biosynthetic pathway and supply of methyl units) and HSP17.8 in wheat leaves [141]. An upregulation in expression of *DREB2A* and *CAT1* was observed during study of gene expression analysed by RT-qPCR in untreated wheat plants however PGPR-inoculated host wheat plants displayed decreased transcript levels indicating enhanced drought tolerance due to rhizobacterial plant interactions [142]. RT-qPCR showed an upregulated expression of *OsDREB1A*, *OsAP37*, *OsNAC6*, *OsGADPH*, *OsWRKY11* and *OsDIL* in rice plants inoculated by consortium of two PGPR strains *Bacillus amyloliquefaciens Bk7* and *Brevibacillus laterosporus B4* which pointed towards MIST to drought [143]. Enhanced drought tolerance was observed in PGPR inoculated wheat plants which was attributed to boosted levels of *TaCTR1* gene and increased expression of *TaDREB2* gene, latter encodes for a transcription factor involved in drought tolerance [144]. Researchers utilized RT-PCR and studied the expression patterns of drought responsive genes in AMF (*Glomus mosseae*) inoculated *Poncirus trifoliata* seedlings and observed elevated mRNA levels of *CSD1*, *MIOX1*, *GlX1* and *TTC1* encoding various antioxidant enzymes involved in maintaining ROS homeostasis and abating oxidative stress in AMF plants in comparison to non AMF plants [145]. A marked increase in expression of genes related to ABA biosynthesis (*ABA1*, *NCED3*, and *ABA3*), signalling (*ABI3*, *ABI4* and *ABI5*) and stress response *(RD22*, *RD29B* and *RAB18A*) was displayed by AMF colonized plants illustrating ABA role in drought tolerance to host plants [48]. Upregulated expression of d-myo-inositol-3-phosphate synthase (IPS) and 14-3GF (related to ABA signal transduction) indicated co-expression of both genes led to crosstalks between host maize plants and AMF enhancing to MIST in plants [127]. AM symbiosis boosted drought tolerance in *Solanum lycopersicum* via regulation of 14-3-3 genes (*TFT1*-*TFT12*) (key genes in ABA signalling pathways) that altered the stomatal behaviour in host plants. An upregulation in expression of *TFT2* and *TFT3* was recorded in AM colonized wild-type (*wt*) which decreased transpiration rate ultimately enhancing WUE of host *wt* plants. Moreover upregulated expression of *TFT5*, *TFT7*, *TFT9* and *TFT10* in AM colonized *not* plants altered transpiration rate subsequently contributing towards better WUE and drought tolerance [146]. Proteome analysis involving mass spectrometry in *Piriformospora indica* inoculated barley plants during drought stress showed raised content of various proteins that are involved in photosynthetic stimulation, redox metabolism or antioxidant defence and energy metabolism hence increasing drought tolerance in host plants [147]. At proteome level, *Glomus masseae* inoculation in wheat roots showed alteration of proteins associated to sugar metabolism, defence responses and cell wall rearrangement. In addition AMF association in durum wheat also showed elevation in 12-oxo-phytodienoic acid reductase and reduced jasmonate-induced protein both correlated to JA synthesis, thus enhancing wheat tolerance to osmotic stress [148]. Two putative genes *LbKT1*, *LbSKOR* (encoding potassium channels) showed positive correlation with potassium contents and colonization rate of AMF in *Rhizophagus irregularis* colonized *Lycium barbarum* ultimately increasing potassium uptake in host plants during drought stress [149]. RT qPCR analysis displayed upregulation of six genes *COX1* (regulates energy and carbohydrate metabolism), *PKDP*, *AP2-EREBP*, *Hsp20*, *bZIP1* and *COC1* in *Pseudomonas fluorescens* strain (*Pf1*) inoculated rice plants during drought stress. *Hsp20*, *bZIP1* and *COC1* are chaperons engaged in (ABA) dependent signalling pathway; *PKDP* functions as protein kinase and *AP2-EREBP* is involved in developmental and stress defensive pathways [150]. *Arabidopsis* plants primed with GAP-P45 showed variation in expression patterns of genes (*ADC*, *AIH*, *CPA*, *SPDS*, *SPMS* and *SAMDC*) related to biosynthesis of polyamines thus enhancing polyamine levels in plants that are crucial to abate osmotic stress during drought [151].

It was observed that the upregulation of root *PtYUC3* and *PtYUC8* genes were related to IAA biosynthesis in *Poncirus trifoliata* inoculated with AMF (*Funneliformis mosseae*) during both well-watered and drought stress conditions [152]. Upregulated transcriptional level of *PtABCB19* and *PtLAX2* (root auxin-species influx carriers) and downregulated level of *PtPIN1* and *PtPIN3* (root auxin efflux carriers) was also noticed during drought stress. Key role of miRNAs in *Pseudomonas putida* RA mediated MIST was studied in chick pea plants [153]. Downregulation of *PtPIN1* and *PtPIN3* led to reduced auxin efflux thereby boosting auxin accumulation in roots ultimately enhancing root hair growth in AM inoculated plants. *GAMYB-like* TF are encoded by genes of miR159 and both were found to be inversely related in AMF treated plants during drought stress. Treated plants displayed upregulated expression of miR159 and downregulated expression of its target *GAMYB-like* TF during drought. A similar inverse relation with downregulation in expression of miR169 and up-regulation in expression of its target, *NF-YA1* responsible for altering expression of various stress genes was observed. Moreover declined expression of miR166 (drought responsive RNA) targeting ATHB15, a representative of Class III homeodomain-leucine zipper was observed that has key role in vascular development and abiotic stress tolerance. A basal expression of miR171 boosted the expression of *NSP2* transcripts (involved in developmental processes and stress signalling pathways) and downregulation of miR167 related to ABA signalling was observed.

Epigenomics, the study to determine entire set of epigenetic alterations that modify genetic material of a cell is the latest omics strategy that can be utilized to study drought tolerance strategies in plants. Epigenetics is the study involving heritable modifications in gene expression comprising epigenetic mechanisms of DNA methylation, histone modifications and small RNAs. Researchers utilized capillary electrophoresis concerted with methylation-sensitive amplification length polymorphism (CE-MSAP) to track DNA cytosine methylation alterations in Burkholderia phytofirmans strain PsJN primed two potato varieties (the Red Pontiac and Superior) [154]. Superior showed enhanced DNA methylation than Red Pontiac indicating correlation between DNA loci methylation and suppression of PsJN mediated plant growth stimulation. There are evidences reporting epigenetic approach to counteract drought stress involving interplay of microbes and host plants. Studies revealed that colonization of Brachypodium by endophyte Bacillus subtilis strain B26 imparted drought tolerance to host plant which was correlated to upregulated expression of stress responsive genes and DNA methyltransferases viz. MET1B-like, CMT3-like and DRM2-like [155]. Enhanced DNA methylation levels and abundance of methyltransferases in colonized host plant were noticed in comparison to non-inoculated plants [156]. The alterations in DNA methylation process led to the conclusion that endophytic colonization impacts the epigenetic regulation of Brachypodium during drought stress. Methyl-sensitive amplified polymorphism (MSAP) was utilized to discover epigenetic modifications associated with drought stress in wheat plants colonized by endophytic fungus SMCD 2206. The DNA methylation patterns recorded in drought stressed wheat seedlings treated with endophyte SMCD 2206 were similar as in unstressed control seedlings. These results revealed that SMCD 2206 inoculation protected wheat seedlings during drought and was associated with the modifications in plant DNA methylation patterns in a similar manner to those shown by non-stressed seedlings [157]. As it was also well demonstrated that AMF colonization of host plant affects the DNA methylation levels [158] hence the future research needs the studies that involves host epigenetic modifications as a result of microbial colonization to improve plant productivity during drought stress. As epigenetic modifications also contribute to stress memory further insights in this direction can help plants to effectively cope with drought stress.

## 5. Compendium

Overall above discussion has led to conclusion that microbial colonization can enhance drought tolerance in host plants that involves various MIST mechanisms. Figure 1 summarizes recapitulation of various MIST mechanisms elicited commonly by AMF and PGPR which systemically encounter the drought stress negative effects and rectify them ultimately improving plant growth and productivity. Drought leads to secondary stresses such as oxidative and osmotic stress which are counteracted via antioxidant armament and osmotic grapnel of microbes respectively. Water deficit reduces photosynthesis in host plants however microbial treated plants display boost in photosynthetic activity due to increased chlorophyll content and higher stomatal conductance. Bacterial EPS, AMF glomalin production, expression of aquaporins and modified RSA due to phytohormonal alterations elevates hydraulic conductivity in plant. In addition, higher nutrient uptake and expression of various stress responsive genes remodels the plant growth during drought stress. Figure 2 sketches drought stress mitigation by AMF and PGPR via modification of various physiological, biochemical and molecular mechanisms. Although there are similarities in the MIST mechanisms of AMF and PGPR however there are slight variations behind the basics of these mechanisms conducted by both. AMF can improve nutrient status of host via boosting CaM levels, hyphal effect and activity of ion transporters, similarly PGPR also raises nutrient content in host plants via siderophore production and nodulation. Enhanced soil aggregation due to glomalin production and modified expression of aquaporins improves water stature in AMF colonized plants. PGPR also contributes to increased hydraulic conductance by improved soil aggregation due to EPS production and modification of root system architecture via hormonal influence such as IAA and ethylene. This improved nutrient and hydraulic conductance alleviates the drought induced growth effects in plants. AMF and PGPR can alleviate osmotic and oxidative stress by altering osmolyte content and antioxidant enzymes in plants. In addition, stomatal activity is altered in response to phytohormones such as ABA, SA (produced by both AMF and PGPR), cytokinins and VOC’s (PGPR). Increased stomatal conductance along with chlorophyll content enhancement contributes to boost in photosynthesis necessary for plant performance during drought.

## 6. Future Prospects

To summarize, microbial inoculated plants employ various MIST mechanisms to counteract drought stress thus ensuring enhanced crop productivity. Considerable research studies has been done to understand the interplay between microbes and plants to enhance MIST to drought in plants, however some aspects still needs to be investigated such as decoding hidden metabolic pathways behind MIST. In future research studies can be focussed on how nutritional uptake affects the various transport channels in microbial inoculated plants and how this can be related to phytohormones such as ABA and brassinosteroids which affect stomatal regulation. In addition studies should be focussed on unravelling the PIN family functions inducing root hair modifications via microbial inoculation under drought conditions.
